# Protective effects of voltage-gated calcium channel antagonists against zinc toxicity in SN56 neuroblastoma cholinergic cells

**DOI:** 10.1371/journal.pone.0209363

**Published:** 2018-12-20

**Authors:** Marlena Zyśk, Beata Gapys, Anna Ronowska, Sylwia Gul-Hinc, Anna Erlandsson, Adam Iwanicki, Monika Sakowicz-Burkiewicz, Andrzej Szutowicz, Hanna Bielarczyk

**Affiliations:** 1 Department of Laboratory Medicine, Medical University of Gdansk, Gdansk, Poland; 2 Department of Public Health & Caring Sciences/Molecular Geriatrics, Uppsala University, Uppsala, Sweden; 3 Department of Molecular Bacteriology, University of Gdańsk & Medical University of Gdańsk, Gdansk, Poland; 4 Department of Molecular Medicine, Medical University of Gdansk, Gdansk, Poland; Doheny Eye Institute/UCLA, UNITED STATES

## Abstract

One of the pathological site effects in excitotoxic activation is Zn^2+^ overload to postsynaptic neurons. Such an effect is considered to be equivalent to the glutamate component of excitotoxicity. Excessive uptake of Zn^2+^ by active voltage-dependent transport systems in these neurons may lead to significant neurotoxicity. **The aim of this study** was to investigate whether and which antagonists of the voltage gated calcium channels (VGCC) might modify this Zn^2+^-induced neurotoxicity in neuronal cells. **Our data** demonstrates that depolarized SN56 neuronal cells may take up large amounts of Zn^2+^ and store these in cytoplasmic and mitochondrial sub-fractions. The mitochondrial Zn^2+^ excess suppressed pyruvate uptake and oxidation. Such suppression was caused by inhibition of pyruvate dehydrogenase complex, aconitase and NADP-isocitrate dehydrogenase activities, resulting in the yielding of acetyl-CoA and ATP shortages. Moreover, incoming Zn^2+^ increased both oxidized glutathione and malondialdehyde levels, known parameters of oxidative stress. In depolarized SN56 cells, nifedipine treatment (L-type VGCC antagonist) reduced Zn^2+^ uptake and oxidative stress. The treatment applied prevented the activities of PDHC, aconitase and NADP-IDH enzymes, and also yielded the maintenance of acetyl-CoA and ATP levels. Apart from suppression of oxidative stress, N- and P/Q-type VGCCs presented a similar, but weaker protective influence. In **conclusion**, our data shows that in the course of excitotoxity, impairment to calcium homeostasis is tightly linked with an excessive neuronal Zn^2+^ uptake. Hence, the VGCCs types L, N and P/Q share responsibility for neuronal Zn^2+^ overload followed by significant energy-dependent neurotoxicity. Moreover, Zn^2+^ affects the target tricarboxylic acid cycle enzymes, yields acetyl-CoA and energy deficits as well.

## Introduction

Zn^2+^ is an essential trace metal playing a regulatory role in diverse cell functions including gene expression, neurotransmision or being a co-factor of over 300 metalloproteins [1–3]. Over 80% of intracellular Zn^2+^ is complexed with metalloproteins, while the remaining non-complexed Zn^2+^ is considered to be nontoxic [1–7]. Our body contains approximately 2 g of Zn^2+^ occurred mostly in muscle, liver and the Zn^2+^-richest brain tissues. In which, the highest brain’s zinc levels were found in the hippocampus, amygdala, thalamus and cortex regions [1–3, 8]. It has been proven that Zn^2+^ inter- and intracellular redistributions in postsynaptic neurons of gluzinergic synapses are essential for proper learning and memory storage processes, taking place in hippocampus [9]. On the other hand, the other data demonstrate that prolonged depolarization of presynaptic gluzinergic terminals may cause an excessive postsynaptic Zn^2+^ uptake, which may trigger the onset of neurodegeneration [6–7, 10–18]. Excessive depolarization disrupts the ionic homeostasis being a primary signal yielding mitochondrial dysfunctions and energy production failure [4, 10–13, 19–23]. Several pathological conditions may cause prolonged depolarization of glutaminergic neurons, which exert excitotoxic effects on postsynaptic neurons through excessive co-release of glutamate and Zn^2+^ [4–5, 11, 24]. Our past studies revealed that the excessive accumulation of Zn^2+^ by SN56 septal cholinergic neuronal cells decreased their acetyl-CoA level and suppressed its utilization in pathways of N-acetylaspartate, ATP, and acetylcholine synthesis [7, 12–13]. There are indications that such Zn^2+^ overloading of postsynaptic neurons may take place *via* inward Zn^2+^ transporters and voltage-gated calcium channels (VGCCs) [4, 19, 21]. Cholinergic SN56 clonal cells, like primary neurons, were found to express all classes of VGCCs [25]. Therefore, this cell line appeared to be a suitable model for studies of participation of Ca^2+^-channels in Zn^2+^-evoked cytotoxicity in cholinergic neurons.

Therefore, the overall goal of this work was to investigate whether and which antagonists of the VGCCs might modify Zn^2+^-induced neurotoxicity in the neuronal cells. Thereby, such information would reveal the contribution of individual VGCC to the early stages of Zn^2+^-evoked excitotoxic neuronal injury.

## Materials and methods

### Materials

Specified reagents and cell culture growth media were obtained from Sigma Aldrich (Poznan, Poland). Antibodies were derived from ABCAM (Straszyn, Poland). TSQ and Western Blot components were derived from Thermofisher (Warszawa, Poland). Cell culture disposables were purchased from Sarstedt (Stare Babice, Poland).

### Cell cultures

**Fig 1:**
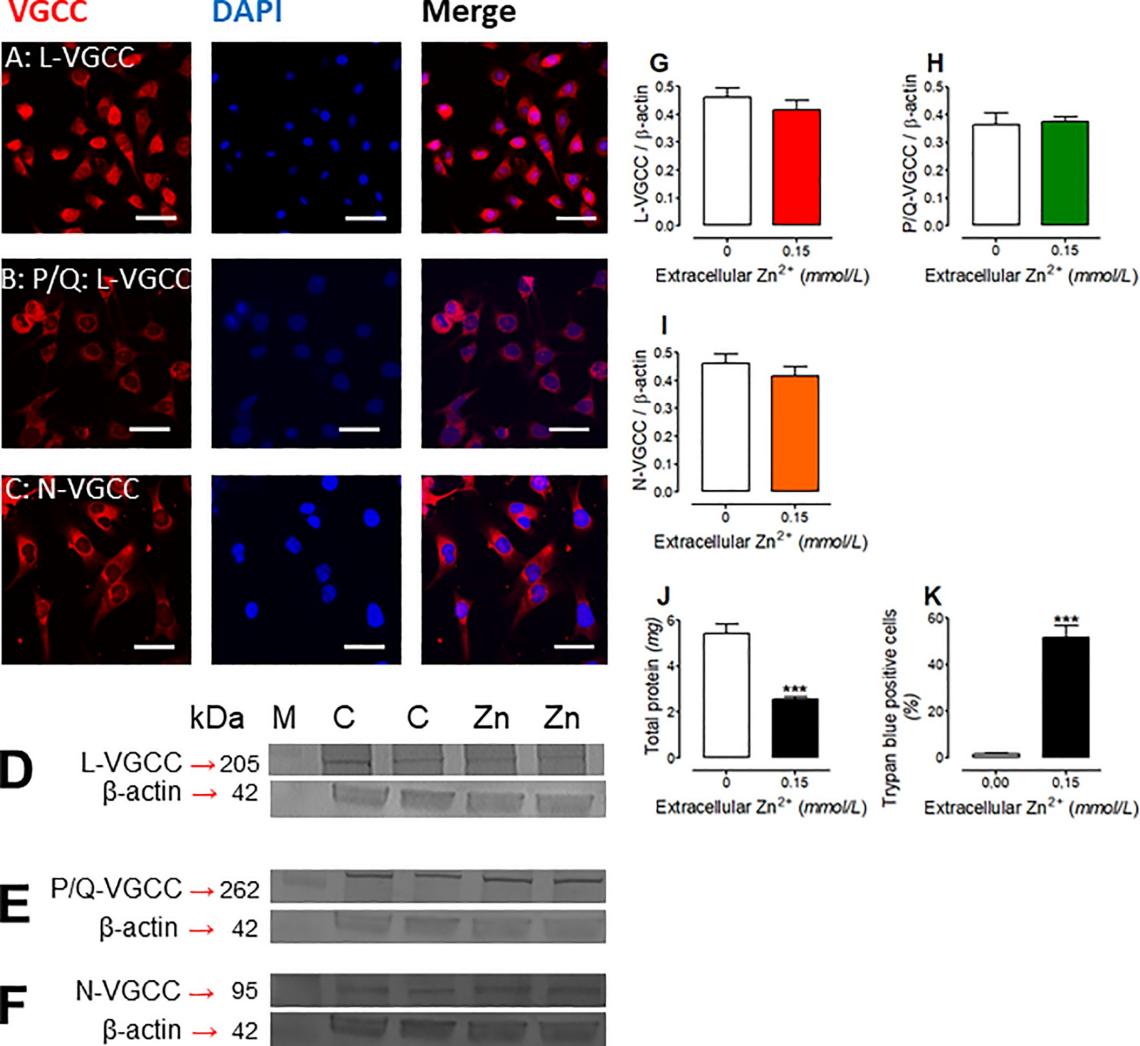
The chronic effect of 0.15 mmol/L Zn^2+^ on SN56 cells. Immunostainings with specific antibodies: (A) L-VGCC, (B) P/G-VGCC, (C) N-VGCC, white bar equals 20 μm. Photographs of Western blot membranes: (D) L-VGCC, (E) P/G-VGCC, (F) N-VGCC as well as the quantitation of the band intensity: (G) L-VGCC, (H) P/G-VGCC, (I) N-VGCC. The confirmation of Zn^2+^ toxicity: (J) total protein level, (K) trypan blue test. Data are means ± SEM from 4–7 experiments. Significantly different from SN56 control (***p<0.0001).

SN56 cells (neuroblastoma cell line SN56.B5.G4) were seeded with a density 40 000 cells/cm^2^ and cultured for 48 h in Dulbecco’s modified Eagle medium containing 2 mM L-glutamine, 0.05 mg of streptomycin and 50 U of penicillin per 1 mL and 10% fetal bovine serum (FBS). For chronic Zn^2+^ toxicity and VGCCs density studies (Fig 1), SN56 cells were cultured for an additional 24h in fresh FBS-DMEM media with or without 0.15 mmol/L Zn^2+^. Such Zn^2+^ concentration was consistent with cytotoxic Zn^2+^ concentrations used in our previous projects [7, 12–13].

A growing body of evidence emphasizes that neurons might actively remove Zn^2+^ ions from their cytoplasm [26–27]. *In vitro* studies showed that such a process requires the physiological concertation of sodium ion concentration and seems to be the most effective after 30 min of *in vitro* incubation [27]. Since one the main goal of acute studies was to determinate the highest capacity of SN56 cells for uptake Zn^2+^, thus cells were placed for 30 min in depolarizing media containing 30 mM K^+^.

For acute Zn^2+^ toxicity studies, SN56 cells were harvested to 320 mmol/L sucrose. The basic, FBS-free depolarizing medium in the final volume of 1 mL contained: 20 mmol/L sodium-HEPES (pH 7.4), 90 mmol/L NaCl, 30 mmol/L KCl, 1 mmol/L CaCl_2_, 1.5 mmol/L Na/K-phosphate buffer (pH 7.4), 32 mmol/L sucrose, 2.5 mmol/L pyruvate, 2.5 mmol/L L-malate, and cell suspension (2–3 mg protein/vessel) [13]. The SN56 cells were pre-incubated for 10 min in media containing VGCC (voltage-gated calcium channel) antagonists at 37°C with shaking of 100 cycles/min. The VGCC antagonists 0.01 mmol/L nifedipine (L-type), 0.0005 mmol/L ω-conotoxin-GVIA (N-type) or 0.0002 mmol/L ω-conotoxin MVIIC (P/Q-type) [28–30]. Each concentration of antagonists was chosen according to its influence on intracellular Ca^2+^ content and Zn^2+^ accumulation (S1 Fig). Next, as part of controls, the cells were treated with 0.05–0.2 mmol/L Zn^2+^. Eventually, after an additional 30 min of incubation, the experiments were terminated by rapid distribution of the SN56 cell suspensions to secondary tubes for different assays (see below).

For Zn^2+^, Ca^2+^, pyruvate, lactate and acetyl-CoA assays, the medium was centrifuged and cell pellets were deproteinized with 4% HClO_4_ and neutralized with 7.5 N K_2_CO_3_. For enzyme activity studies, cell pellets were re-suspended in 320 mmol/L sucrose and lysed with 0.2%.

### Cell viability assay

Cells viability was assessed with trypan blue exclusion assay as described elsewhere [12–13]. In brief, 2.5 mln cells/1mL were dyed by 0.2% Trypan Blue solution (10 min, room temperature). Next, 20 μL of cell suspension was analyzed by counting chamber under light microscope (Axiovert 25, Zeiss). Non-viable cell was identified as unable to remove applied dye.

### Zn^2+^ and Ca^2+^ assays

Intracellular Ca^2+^ levels were assayed with the Arsenazo III spectrophotometric method [31]. Briefly, 1.0 mL of reaction buffer contained 60 mM MOPS buffer (pH = 7.4), 0.8 μM arsenazo III, 45 mM KCl, neutralized supernatant (100 μg of cell homogenate protein) was incubated for 5 min (room temperature). Achieved absorbance (λabs = 650 nm) was counted according to the standard curve values (2–25 nmol).

Zn^2+^ levels were measured using a modified N-(6-methoxy-8-quinolyl)-p-toluenesulfonamide (TSQ) fluorimetric method [32]. Briefly, 1.6 mL of reaction buffer contained 0.3 mM sodium-HEPES (pH = 7.4) and 2 mg/mL TSQ. Fluorometric assay was initiated by 0.2 mL of neutralized supernatant (100 μg of cell homogenate protein) and reaction was carried out at room temperature for next 20 min (λex = 335 nm, λem = 495 nm). The maximal emission was counted according to the standard curve relative fluorescence units (0.2–4 nmol).

Isolation of mitochondria was carried out for 30 sec in mitochondrial isolation buffer (0.14 mg/mL digitonin, 125 mM KCl, 20 mM HEPES (pH = 7.4), 3 mM EDTA) then layered on AR20:AR200 oil mixture (1:2) and finally spun down (30 sec, 14 000 x g) [33]. The purity of the mitochondrial fraction, controlled by citrate synthase and lactate dehydrogenase activities, was over 90% [34].

### Pyruvate and lactate assays

Rates of pyruvate utilization and lactate accumulation were calculated from differences in metabolite contents in the whole medium at the times zero and after 30 min of incubation [35]. Lactate and pyruvate levels were determined using NADH/NAD conversion technique, at 340 nm and 37°C. Both assays were initiated by the 10 μL addition of 4 U lactate dehydrogenase (EC 1.1.1.27).

1 mL of reaction buffer for lactate assay contained 70 mM glycine-NaOH buffer (pH = 10), 5 mM NAD and 100 μg of cell homogenate protein in a final volume of 0.7 mL.

1 mL of reaction buffer for pyruvate assay contained 0.1 M TRIS-HCl (pH = 7.4), 3 mM sodium-EDTA, 0.2 mM NADH and 100 μg of cell homogenate protein.

### Acetyl-CoA assay

Acetyl-CoA level was assayed in a cell pellet using the cycling method as described elsewhere [12–13, 33]. Briefly, in each neutralized supernatant (40 μg of cell homogenate protein) following reactions were performed: (1) Coenzyme-A removal, (2) acetyl-CoA level enhancement, (3) citrate level measurement. First reaction (1) was carried out in 50 μL for 2 h (room temperature, gentle shaking) with reaction buffer contained 0.1 M TRIS-HCl (pH = 7.4), 1 mM maleic anhydride (dissolved in diethyl ether). Second reaction was starting by the 50 μL addition of second reaction buffer (50 mM TRIS-HCl (pH = 7.4), 5 mM NH_4_Cl, 0.01% albumin, 1.2 mM oxaloacetate, 2 mM acetyl phosphate, 1 U phosphotransacetylase 0.12 U citrate synthase). 100 min lasting reaction (30°C, gentle shaking) was terminated by thermic shock (10 min, 100°C). Finally the produced citrate level was determined using NADH/NAD conversion technique, at 340 nm and 37°C. 0.7 mL of reaction buffer contained 0.1 M TRIS-HCl pH = 7.4, 0.1 mM NADH, 0.2 U malate dehydrogenase (EC 1.1.1.37) and analyzed supernatant. The assay was initiated by the 10 μL addition of 0.1 U citrate lyase (EC 4.1.3.6).

### ATP and ADP assay

ATP and ADP levels were assayed with a modified HPLC method [36], using a mobile phase A: 2.8 mmol/L tetrabutylammonium hydroxide, 25 mmol/L KH_2_PO_4_ and 1.25% methanol, pH 7.00; and a mobile phase B: 100% methanol (flow rate: 0.9 mL/min). Gradient program: 40 min (99% phase A), 60 min (from 99% to 60% of phase A), 5 min (from 99% to 60% of phase A), 20 min (99% phase A). Retention times were: t_ADP_ = 68.1 min t_ATP_ = 79.2 min.

### GSH and GSSG assay

Cell pellets were homogenized for 30s and lysed for 30 min in 0.008 mmol/L N-ethylmaleimide / 5% meta-phosphoric acid (4°C) and neutralized with 2M Tris [37]. After that, each supernatant was divided for “reduced glutathione” and “total glutathione” assays. For “total gluthathione”, the obtained supernatant was incubated for 20 min with 0.17 mmol/L DTT (room temperature). Next, both samples were assayed with the isocratic HPLC method (Perkin Elmer A-200 system) using mobile phase: 2.8 mmol/L tetrabutylammonium hydroxide, 25 mmol/L KH_2_PO_4_ and 1.25% methanol, pH 7.00 (flow rate: 0.9 mL/min). Retention time: t_GSH_ = 4.7 min. GSSG levels were calculated as the difference between total GSH and GSH levels [36–37].

### TBARS assay

The lipid peroxidation products were estimated in the whole medium as thiobarbituric acid reactive substances (TBARS) at 535 nm in room temperature [38]. Briefly, 0.5 mg of cell homogenate protein was deproteinized by 10% trichloroacetic acid in a final volume of 0.6 mL (10 min, 4°C, gentle shaking). Next, each sample was enriched by 0.2 mL of 2% thiobarbituric acid and heated for 20 min in 100°C.

### Enzymes assays

The activities of pyruvate dehydrogenase complex (EC 1.2.4.1), aconitase (EC 4.2.1.3), NADP-isocitrate dehydrogenase (EC 1.1.1.42) were assayed after lysis of the cell pellet in 0.2% triton X-100 as described elsewhere [12–13].

Isocitrate dehydrogenase (IDH-NADP, EC 1.1.1.42) activity was determined using NADPH/NADP conversion technique, at 340 nm and 37°C. The reaction buffer contained 0.05 M TRIS-HCl (pH = 7.4), 0.6 mM MgCl_2_, 0.5 mM NADP and 100 μg of cell homogenate protein in a final volume of 0.7 mL. Enzymatic assay was initiated by the 10 μL addition of 10 mM isocitrate.

Aconitase (EC 4.2.1.3) activity was determined using NADPH/NADP conversion technique, at 340 nm and 37°C. The reaction buffer contained 0.05 M TRIS-HCl (pH = 7.4), 2 mM MgCl_2_, 0.1 mM NADP, 1 U IDH-NADP and 100 μg of cell homogenate protein in a final volume of 0.7 mL. Enzymatic assay was initiated by the addition of 10 mM cis-aconitane (10 μL).

Pyruvate dehydrogenase complex (PDHC, EC 1.2.4.1.) activity was determined using cycling method. In each lysate (100 μg of cell homogenate protein) following reactions were performed: (1) citrate production, (2) citrate level measurement. First reaction (1) was carried out in 250 μL for 30 min (37°C, gentle shaking), buffer contained 0.1 M TRIS-HCl (pH = 8.3), 2 mM MgCl_2_, 10 mM dithiotreitol, 10 mM pyruvate, 2 mM thiamine pyrophosphate, 0.2 mM CoA, 2.5 mM oxaloacetate, 2 mM NAD, 0.15 U citrate synthase (EC 4.1.3.7). Reaction was terminated by thermic shock (10 min, 100°C). Finally the produced citrate level was determined using NADH/NAD conversion technique, at 340 nm and 37°C. The reaction buffer contained 0.1 M TRIS-HCl (pH = 7.4), 0.1 mM NADH, 0.2 U malate dehydrogenase (EC 1.1.1.37) and 100 μL of achieved supernatant in a final volume of 0.7 mL. The assay was initiated by the 10 μL addition of 0.1 U citrate lyase (EC 4.1.3.6).

### Western Blot analysis

SN56 cells were lysed with a RIPA buffer (50 mmol/L TRIS-HCl buffer (pH 7.4), 5 mmol/L EDTA, 100 mmol/L NaCl, 1% Triton-X100, 5% glycerol, 10 mmol/L KH_2_PO_4_) supplemented with a protease inhibitor cocktail. Supernatants were boiled for 5 min with a 2.5% β-mercaptoethanol/Laemmli buffer and then loaded on 4–20% gradient SDS-PAGE gels. The obtained proteins were transferred to a PVDF membrane. Next, the membrane was incubated with primary antibodies in a WB buffer (5% bovine serum albumin/0.5% Tween20/ 25 mmol/L Tris-buffered saline, pH 7.4) at 4°C, overnight. The following day, a after 3-time washing step, the PVDF membrane was incubated for 3h with an alkaline phosphatase-conjugated secondary antibody at room temperature (in a WB buffer). The PVDF membrane was developed using 5-bromo-4-chloro-3-indoylphosphate with Nitro blue Tetrazolium chloride. β-actin was used as a reference protein.

### Immunocytochemistry

SN56 cells grown on cover slips were fixed in 4% paraformaldehyde for 15 min followed by a 60 min blocking step in 5% normal goat serum/0.3% Triton-X100/PBS (room temperature). Then, the fixed cells were incubated overnight at 4°C with primary antibodies diluted in 0.5% normal goat serum/0.03% Triton-X100/PBS (anti-L type VGCC 1:200, anti-N type VGCC 1:200, anti-P/Q type VGCC 1:100). On the following day, after 3x10 min washing steps (0.3% Triton-X100/PBS), the cover slips were incubated for 3 h with rhodamine-conjugated secondary antibodies and 5 min with DAPI solution (0.5 μg/mL). A fluorescence microscope (Observer Z1 Zeiss, Germany) was used for microphotograph capture.

### Protein assay

Protein levels were assayed using the Bradford method [39] with human immunoglobulin as a standard.

### Statistics

Statistical differences between the two groups were assessed with the Mann-Whitney U test and for multiple comparisons the Kruskal-Wallis test with Dunn’s *post hoc* test was used. Values of *p*<0.05 were considered statistically significant. The data presented are means ±SEM from 4 to 25 experiments.

## Results

### Effect of Zn^2+^ on voltage gated calcium channels in SN56 cells

Excessive release of Zn^2+^ from pathologically depolarized glutaminergic terminals was identified as an important factor triggering early neurodegenerative events in postsynaptic neurons [11, 23, 40]. Our past studies on SN56 cells revealed that cholinotoxicity may be partially mediated by depolarization–dependent Zn^2+^ uptake [7, 12–13]. However, no specific channel type has been identified yet. Here, we demonstrate, using specific antibodies, that the plasma membranes of SN56 cells contain the L, P/Q and N types of VGCCs (Fig 1A–1C). Our double staining with DAPI and specific antibody against L-VGCC, P/Q-VGCC as well as N-VGCC revealed that all viable SN56 cells were positive for studied channels (Fig 1A–1C).

One-day treatment of SN56 cells with 0.15 mmol/L Zn^2+^, in a non-depolarizing culture medium, reduced both cell viability and total protein by about 50% (Fig 1J and 1K). However, in cells which were subjected to this treatment no changes in the levels of any of the three VGCC proteins took place (Fig 1D–1I).

### Intraneuronal Zn^2+^ accumulation

**Fig 2:**
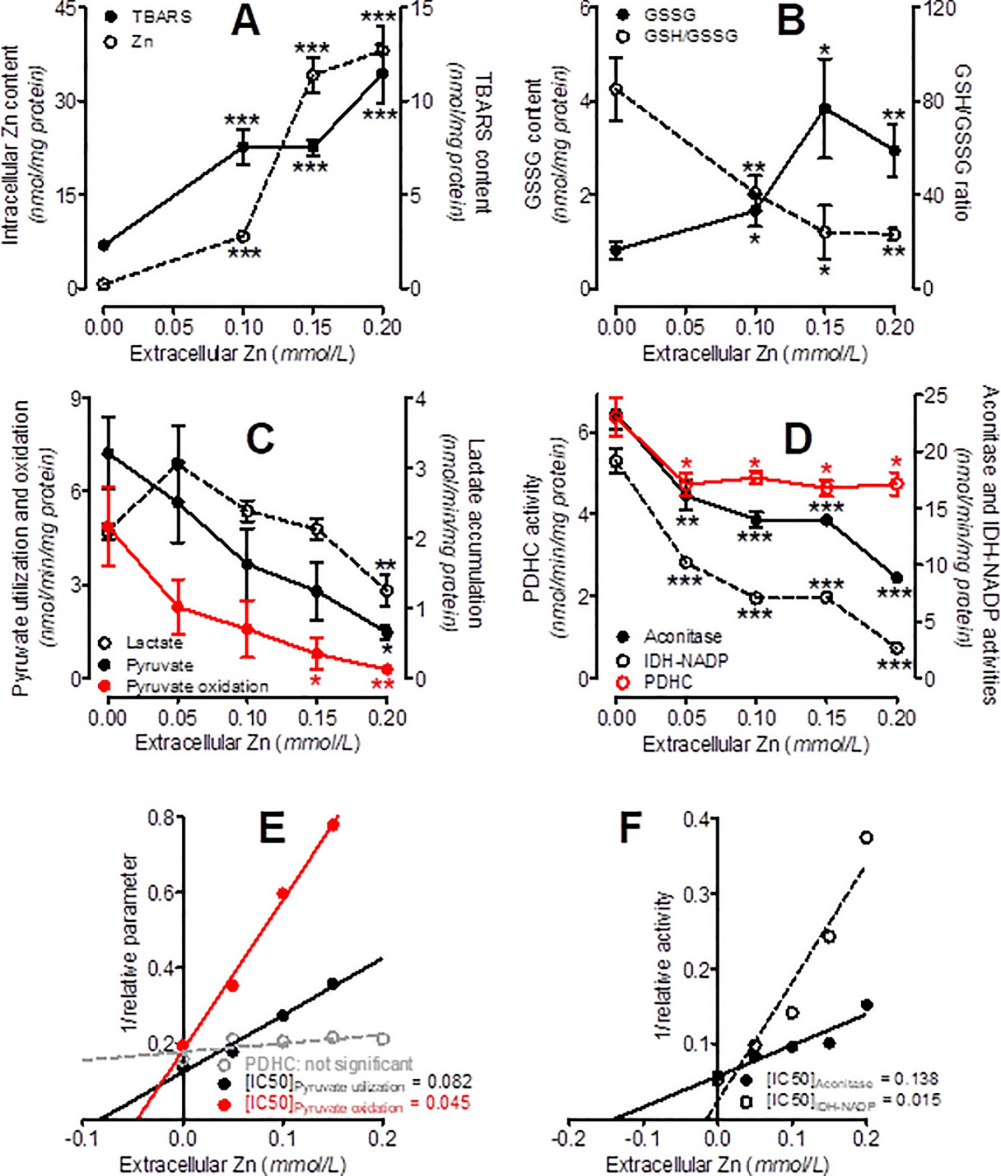
Effects of acute treatment of SN56 cells with increasing Zn^2+^ concentrations. (A) Zn^2+^ accumulation and TBARS content, (B) glutathione oxidation, (C) pyruvate and lactate activities, (D) enzyme activities, (E) Dixon plots calculated from panels 2C and 2D, (F) Dixon plots calculated from Fig 2D. Data are means ± SEM from 4–25 experiments. Significantly different from SN56 control (*p<0.05, **p<0.01, ***p<0.0001).

The control level of intracellular Zn^2+^ in SN56 cells incubated for 30 min in an FBS free and Zn^2+^-free depolarizing medium was equal to 0.6–0.8 nmol/mg protein (Fig 2A, Table 1). In these conditions, the addition of 0.10 and 0.15 mmol/L Zn^2+^ increased the intracellular content of Zn^2+^ to about 8 and 34 nmol/mg protein, respectively (Fig 2A). Furthermore, the acute accumulation of Zn^2+^ in SN56 cells was 2.6 times higher than that found in those cells treated with Zn^2+^ chronically in a non-depolarizing medium containing 10% FBS (Table 1). However, irrespective of the treatment conditions, the general patterns of Zn^2+^ subcellular distribution were similar. Particularly, in SN56 cells, we observed minor mitochondrial and larger cytoplasmic pools, containing 12–20 and 88–80% of the whole cell content, respectively (Table 1). The short-term treatment of depolarized SN56 cells with 0.15 mmol/L extracellular [Zn^2+^], was accompanied by a relatively smaller, 30% elevation of intracellular Ca^2+^ levels (Table 1).

**Table 1:** Subcellular distribution of Zn^2+^ in SN56 cells after 30 min and 24h treatment with this cation.

Incubation time	Addition Zn^2+^ 0.15 mmol/L	Whole cells nmol/mg protein	Mitochondria nmol/mg protein	Cytoplasm nmol/mg protein
**30 min, K^+^ 30 mmol/L^a^**	-	0.64 ±0.1	0.10 ±0.05	0.50 ±0.2
	+	32.1 ±1.6***	3.5 ±0.7***	26.5 ±1.1***
**24 h, K^+^ 5 mmol/L^b^**	-	2.2 ±0.8	0.03 ±0.01	2.1 ±0.2
	+	12.2 ±0.8***^,†††^	2.5 ±0.2***	10.8 ±0.8***^,†††^

a: FBS-free depolarizing media

b: 10% FBS-DMEM non-depolarizing media.

Data are means ± SEM from 3–7 experiments. Significantly different from: respective Zn^2+^-free control (***p<0.0001); or Zn^2+^ from respective 30 min of treatment (^†††^p<0.001).

### Effects of Zn^2+^ uptake on energy metabolism in SN56 cells

**Fig 3:**
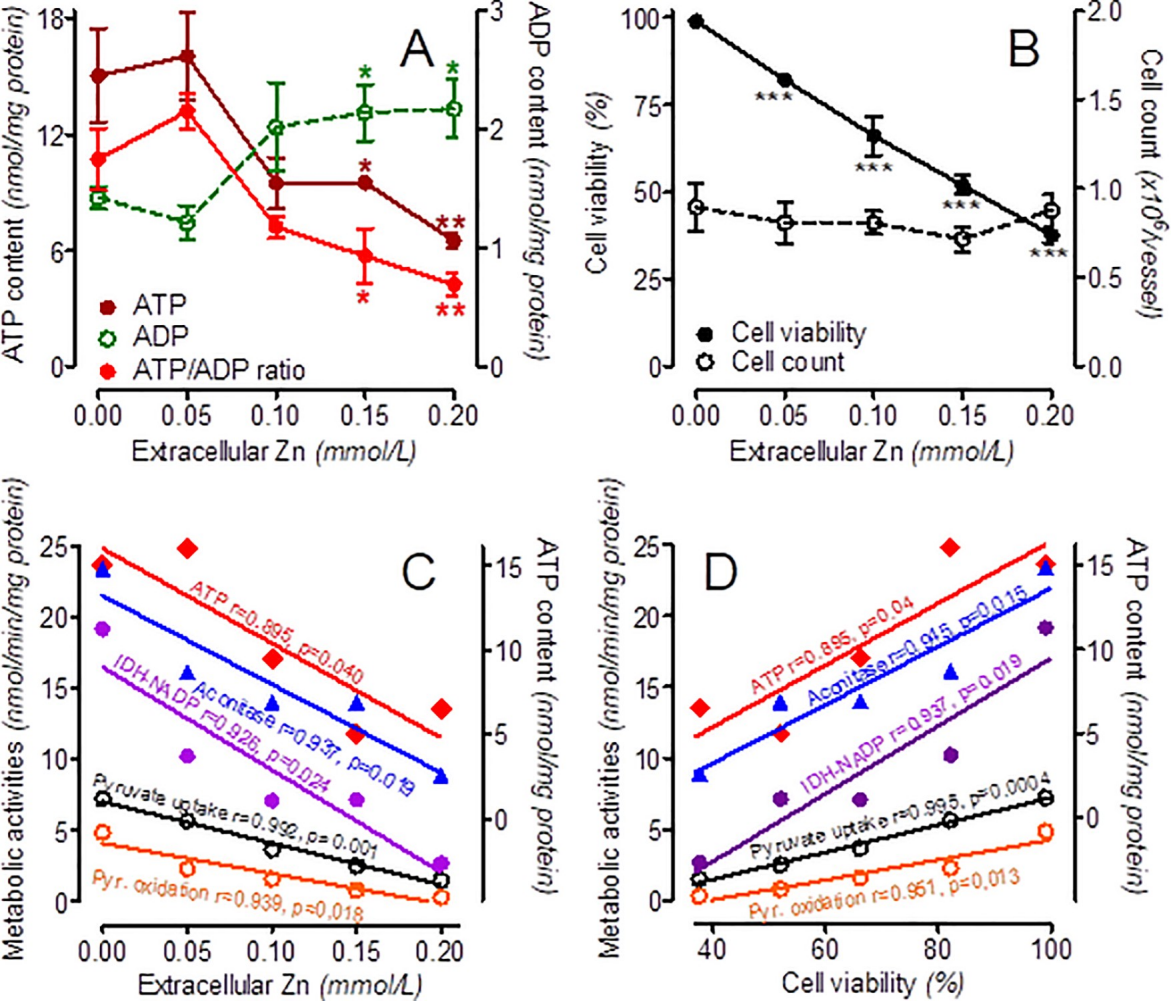
Effects of short-term treatment of SN56 cells with increasing Zn^2+^ concentrations. (A) ATP, ADP levels and ATP/ADP ratio, (B) cell viability and counts, (C) correlation plots of ATP levels and metabolic activities with extracellular Zn^2+^ concentration, (D) correlation plots of ATP levels and metabolic activities with fractional cell viability. Data are means ± SEM from 3–6 experiments. Significantly different from SN56 control (*p<0.05, **p<0.01, **p<0.0001).

The concentration-dependent Zn^2+^ accumulation in SN56 cells brought about, progressing up to three-fold, an increase of TBARS synthesis, a 4.5-fold rise of GSSG level, and a 70% decrease of the GSH/GSSG ratio (Fig 2A and 2B) These changes were accompanied by dose-dependent inhibition of pyruvate utilization by SN56 cells, from 6.4 in controls to 3.4 nmol/min/mg protein at 0.15 mmol/L extracellular Zn^2+^ (Figs 2C, 2E and 3C). Meanwhile, lactate accumulation decreased from 2.1 to 1.2 nmol/min/mg protein (Fig 2C). The rate of pyruvate oxidation, calculated as the difference between pyruvate uptake and lactate formation, decreased from 4.1 to 0.4 (Fig 2C). Zn^2+^ [IC_**50**_] for pyruvate utilization was equal to 0.082 mmol/L, which was nearly two times higher than [IC_**50**_] for pyruvate oxidation (Fig 3D). On the other hand, PDHC activity was inhibited only by 25% and revealed no dependence on metal concentration (Fig 2D and 2E).

The activities of aconitase and IDH-NADP were inhibited by accumulating Zn^2+^ in a concentration-dependent manner (Fig 2D and 2F, Table 2). Zn^2+^ [IC_**50**_] for IDH-NADP was equal to 0.015 mmol/L and appeared to be 9 times lower than that of aconitase (Fig 2D and 2F). In the same conditions, a 55% reduction of the ATP level at a 50% elevation of the ADP level caused a respective decline of the ATP/ADP ratio (Fig 3A, 3C and 3D, Table 2). These short-term alterations did not change the total cell number, but decreased the viable trypan blue excreting cell fraction by 40% (Fig 3B, Table 2).

**Table 2:** VGCC antagonists modify the acute Zn^2+^ neurotoxic effects on K^+^ depolarized SN56 cholinergic neuroblastoma cells.

Parameter	Addition Zn^2+^ 0.15 mmol/L	Control	Nifedipine 0.01 mmol/L	GVIA 0.0005 mmol/L	MVIIC 0.0002 mmol/L
**Zn**^**2+**^ **level** *nmol/mg protein*	-	0.6 ±0.1	0.6 ±0.1	0.8 ±0.3	0.7 ±0.1
	+	34.1±2.7***	13.8±2.0***^†††^	18.7±0.8***^††^	16.1±0.7***^†††^
**Ca^2+^ level** *nmol/mg protein*	-	24.4 ±1.1	16.4 ±1.9^†††^	20.1 ±0.5^†††^	21.3 ±2.2
	+	31.5 ±2.4***	22.7 ±2.4^†^	19.0 ±0.5^†††^	20.6 ±1.2^††^
**TBARS level** *nmol/mg protein*	-	2.3 ±0.1	1.6 ±0.2	3.1 ±0.3	2.8 ±0.1
	+	7.5 ±0.4***	2.9 ±0.3***^†††^	6.6±0.4***^‡‡‡^	5.5 ±0.2***^†‡‡^
**Trypan blue positive cells** *%*	-	3.4 ±0.7	3.8 ±0.7	1.3±0.4	2.0 ±0.5
	+	40.0 ±2.9***	17.1±2.9***^†††^	31.4±3.7***^†‡‡^	33.0±2.3***^†‡‡^
**Acetyl-CoA level** *pmol/mg protein*	-	30.5 ±0.6	30.7 ±1.0	28.8 ±1.6	28.1±3.1
	+	13.8 ±0.3***	29.2 ±0.7^†††^	21.6 ±2.7*^†††‡^	20.5±1.7^†††‡‡^
**ATP level** *nmol/mg protein*	-	15.1 ±2.4	14.9 ±1.0	N.A.	N.A.
	+	8.5 ±0.8***	12.2 ±0.7***^††‡^	N.A.	N.A.
**Aconitase** relative activity, *%*	-	100.0 ±3.4	94.7 ±3.6	106.3 ±4.0	107.8 ±6.6
	+	56.9 ±3.3***	74.1 ±4.9*^††^	79.0 ±5.0*^††^	77.8 ±4.9**^††^
**IDH-NADP** relative activity, *%*	-	100.0 ±2.8	98.7 ±1.5	100.0 ±3.0	91.3 ±5.1
	+	45.2±3.8***	67.8 ±2.8***^†††^	48.2 ±7.2***^‡‡^	43.4 ±13.8***^‡‡^

Data are means ± SEM from 4–25 experiments. Significantly different from respective: no Zn^2+^ conditions (*p<0.05,**p<0.01, ***p<0.001); no VGCC conditions (^**†**^p<0.05, ^**††**^p<0.01, ^**†††**^p<0.001); 0.01 mmol/L nifedipine conditions (^‡^p<0.05, ^‡‡^p<0.01, ^‡‡‡^p<0.001).

Significant inverse relationships were found between an extracellular Zn^2+^ concentration and the rates of: pyruvate uptake and oxidation, aconitase and IDH-NADP activities, as well as ATP levels in SN56 cells (Fig 3C). On the other hand, the Zn^2+^-evoked decrease of viable cell fraction displayed positive correlations with the declining values of metabolic parameters quoted above (Fig 3D).

### Voltage-gated calcium channel blockers and Zn^2+^ cytotoxicity

The saturating concentrations of voltage-gated calcium channel (VGCC) antagonists were determined experimentally (S1 Fig). In a Zn^2+^-free medium none of the antagonists (0.01 mmol/L nifedipine, 0.0005 mmol/L ω-conotoxin GVIA and 0.0002 mmol/L ω-conotoxin MVIIC) altered the low endogenous level of intracellular Zn^2+^ (Table 2). In the absence of Zn^2+^, these Ca^2+^-antagonists altered neither lipid peroxidation, nor the fraction of non-viable SN56 cells, respectively (Table 2). Furthermore, no significant effects of the antagonists on acetyl-CoA and ATP levels or aconitase and IDH-NADP activities were detected (Table 2). Nifedipine and GVIA decreased cellular Ca^2+^ levels by about 33 and 18%, respectively. The MVIIC exerted no significant effect on intracellular Ca^2+^ content (Table 2). In a medium containing 0.15 mmol/L Zn^2+^, each of those VGCC antagonists reduced its influx into SN56 cells by 45–60% (Table 2). All VGCCs also prevented Zn^2+^-induced excessive accumulation of Ca^2+^ and partially prevented aconitase inhibition (Table 2).

**Fig 4:**
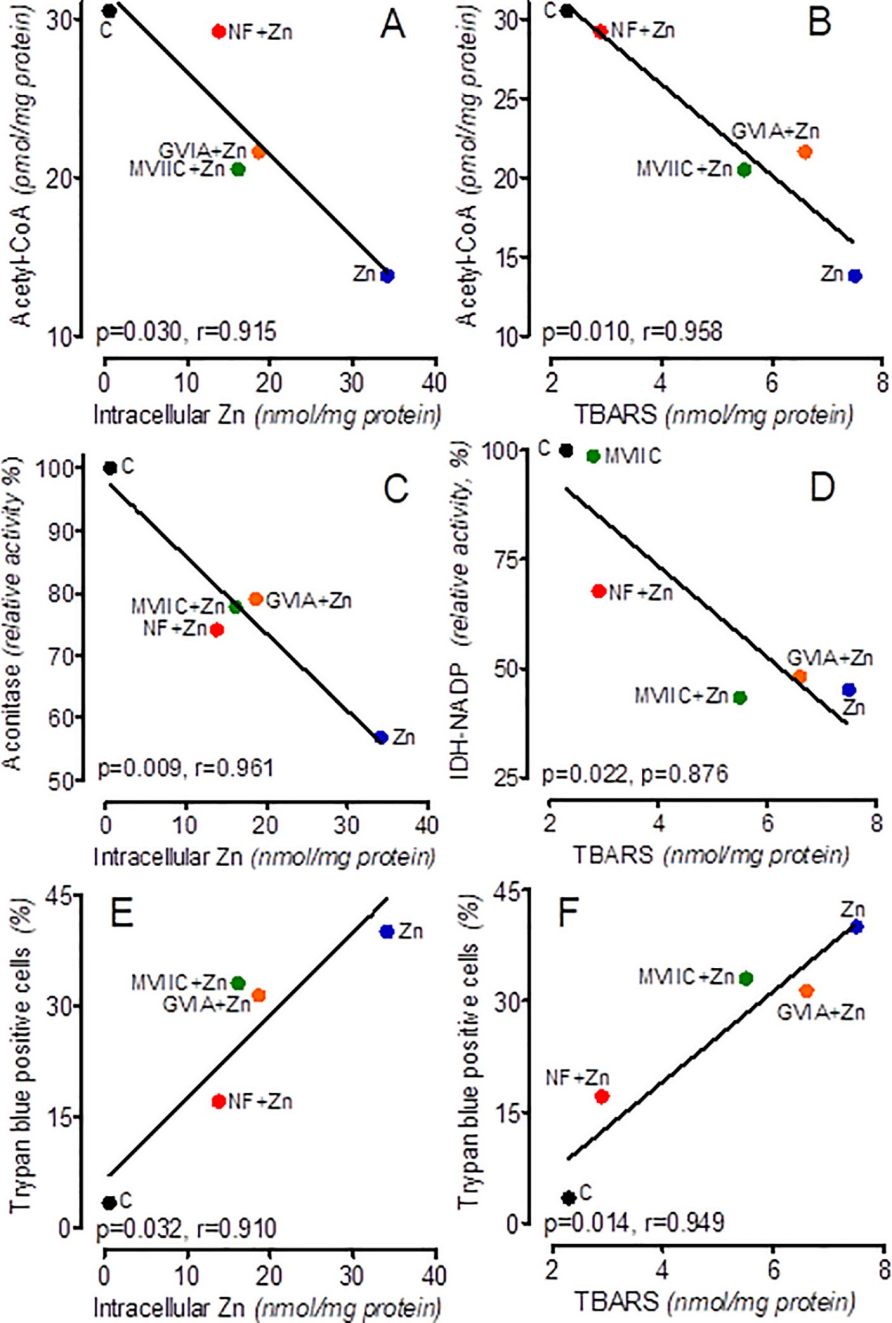
Correlation plots. Correlation plots of acetyl-CoA levels (A), aconitase activity (C), fractional cell injury (E) against intracellular levels of Zn^**2+**^ and acetyl-CoA levels (B), IDH-NADP activity (D), fractional cell injury (F) against TBARS accumulation rate in SN56 cells incubated for 30 min in a depolarizing medium with 0.15 mmol/L Zn^**2+**^ and different VGCC. Plots were calculated from data presented in Table 2.

Despite these similarities, nifedipine exerted the strongest protective effects against Zn^2+^-toxicity, reducing the non-viable fraction from 40 to 17% (Fig 4E and 4F, Table 2). In the presence of nifedipine, Zn^2+^ treatment did not promote TBARS synthesis at fully preserved acetyl-CoA content and partially protected the ATP level as well (Fig 4A and 4B, Table 2). Nifedipine also alleviated the inhibitory effects of Zn^2+^ treatment on both aconitase and IDH-NADP activities (Fig 4C and 4D, Table 2).

Our studies showed that both ω-conotoxins (GVIA and MVIIC) appeared to be weaker neuronal protectants than nifedipine. They reduced the fraction of trypan blue positive SN56 cells to 31 and 34%, respectively (Fig 4E and 4F, Table 2). However, these antagonists did not reduce the excessive synthesis of TBARS and did not augment the inhibition of IDH-NADP activity. GVIA and MVIIC were not fully effective in the maintenance of acetyl-CoA level, increasing it from 45 to 69% of control levels (Fig 4A and 4B, Table 2). These VGCC-induced alterations in reducing inhibitory Zn^2+^ effects on enzyme activities and metabolite levels in SN56 cells correlated significantly with their capacities for reducing intracellular Zn^2+^ overload or/and TBARS accumulation (Fig 4A–4F).

## Discussion

**Fig 5:**
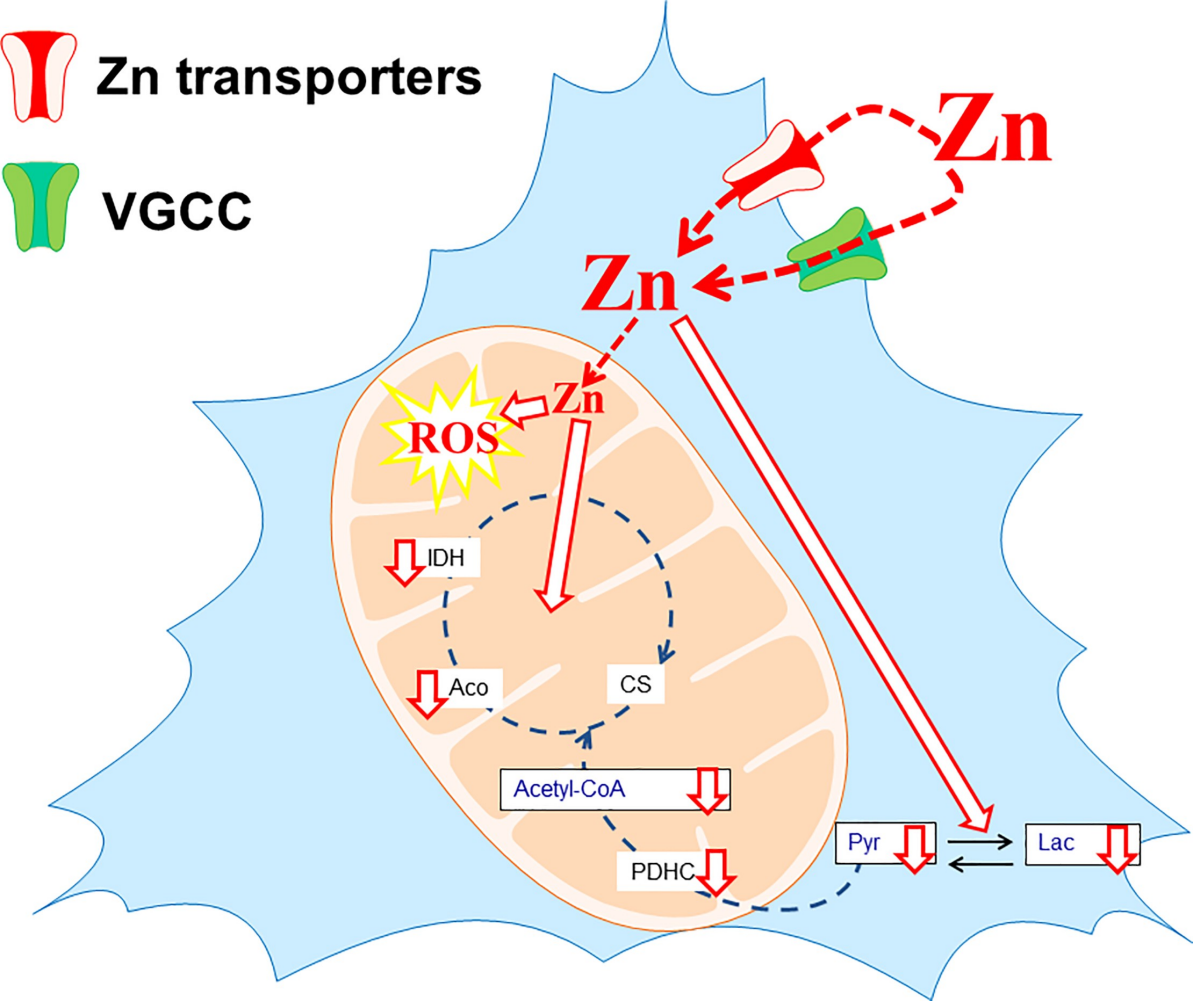
Graphical abstract. Zinc accumulation *via* VGCC and Zn^2+^-transporters might lead to the suppression of PDHC, aconitase and isocitrate dehydrogenase enzymes.

It has been demonstrated that Zn^2+^ accumulation in depolarized postsynaptic neurons *via* diverse transporting systems exerts a negative effect on a several intracellular targets (Fig 5). Its excessive uptake by neurons yields functional and structural impairment, which eventually leads to their death [4–5, 11, 24]. In our cholinergic cellular model of Zn^2+^ neurotoxicity we used pathologically relevant concentrations of Zn^2+^ (0.05–0.20 mmol/L), which correspond well with assessed cation levels in the synaptic cleft and whole brain tissue as well (Figs 2 and 3)[41–42]. The basal Zn^2+^ content in SN56 cell was equal 0.6 nmol/mg protein (0.08 mmol/L of cell water), which is comparable with 0.1–0.2 mmol/L cation levels reported for the whole brain or primary neurons (Tables 1 and 2)[43–46].

It is known that postsynaptic cells take up zinc ions *via* different Zn^2+^- and Ca^2+^- specific channels, although their participation in Zn^2+^ transport is mainly related to neurons [47]. Our immunostainings confirmed the existence of the main neuron-specific calcium channel classes on the surface of clonal cholinergic SN56 cells (Fig 1A–1I). These findings are compatible with patch camp studies revealing the functional existence of these VGCCs in SN56 cells [25]. Thereby, mechanisms of Zn^2+^ overload presented by using the clonal SN56 neuronal cells may be compatible with those reported for *in vivo* and *in vitro* models of brain excitotoxicity-related pathologies [48–49]. Therefore, we conclude that neither short- nor long-term increases of Zn^2+^ at gluzinergic synapses trigger mechanisms of VGCCs down regulation in postsynaptic neurons (Fig 1A–1I). These observations point out that cholinergic neurons may not suppress VGCC expression as a protective mechanism against prolonged increases of Zn^2+^ in the synaptic cleft (Fig 1D–1I). Furthermore, accumulation of Zn^2+^ by chronically exposed SN56 cells in a non-depolarizing medium was about 3 times lower than in those exposed to Zn^2+^ for 30 min in a depolarizing medium (Table 1). These differences in Zn^2+^ levels may result from a depolarization-dependent increase of VGCC permeability for Zn^2+^ [47, 49–51]. Relatively low levels of Zn^2+^ accumulation in mitochondria may indicate that these organelles possesses an effective system of Zn^2+^ excretion to the cytoplasm or/and relevant mechanisms preventing the fast inward transport of Zn^2+^ from the cytoplasm (Table 1)[49, 52–53]. These findings remain in accord with primary neuron studies showing slow mitochondrial uptake of Zn^2+^ *via* Ca^2+^-uniporters or other mitochondrial zinc-specific transporters [49, 52–53]. Nevertheless, this relatively small mitochondrial Zn^2+^ accumulation significantly affected SN56 cell oxidative metabolism (Figs 2C–2F and 3)[51, 54–56]. On the other hand, the activities of cytoplasmic enzymes, such as ATP-citrate lyase or choline acetyltransferase, lactate dehydrogenase, autotaxin and caspase-3, appeared to be resistant to acute influences of high Zn^2+^ concentrations [12–13, 57–58]. Therefore, changes triggered by Zn^2+^ excess in cytoplasmic acetyl-CoA levels should be considered secondary to alterations in its mitochondrial availability (Fig 4A and 4B)[12–13].

The presented data demonstrate that Zn^2+^ might trigger Ca^2+^ overload in SN56 cells (Table 2), which may result from ATP deficits yielding a substrate concentration-dependent decrease of Ca^2+^-ATP-ase activity and augmentation of free radical-evoked impairment of cell viability (Table 2) [10, 49, 59]. Also, the prolonged cell depolarization associated with such conditions may contribute to Ca^2+^ overload due to increased VGCC permeability (Table 2) [16, 19]. In fact, Zn^2+^ caused upregulation of T-type VGCC in hippocampal neurons *in vivo* [50]. Zn^2+^ might also facilitate Ca^2+^ entry through activation of the transient receptor potential channel A1^3+^ [60].

Our past data revealed that Zn^2+^ reversibly inhibits PDHC activity in cell homogenates due to the removal of lipoamide from E2 subunit binding sites [12–13]. Here, we investigated the pyruvate uptake/utilization in whole, not lysed, SN56 cells. Relatively strong inhibition of pyruvate oxidation *in situ*, triggered by Zn^2+^ overload, indicates that its “free”- ion levels reached sufficient concentrations to inhibit PDHC in a similar manner as in homogenates (Fig 2A, 2C and 2D) [13]. Such conditions did not inhibit PDHC, as demonstrated here by unaltered complex activity measured in cell homogenates with saturating concentrations of its substrates and cofactors (Fig 2D and 2E)[12–13]. This indicates that the short-term toxic effects of Zn^2+^ on mitochondrial metabolism of pyruvate in the brain may be partially or fully reversible (Fig 2C and 2E) [6, 12, 20, 61]. Accordingly, an excess of intramitochondrial Zn^2+^ *in vivo* may be the single factor causing transient pyruvate derived acetyl-CoA deficits in excitotoxicity-affected neurons (Figs 2C–2E and 4,Tables 1–2).

It has been shown that the inhibitory effects of Zn^2+^ on aconitase and IDH-NADP activities in SN56 homogenates were irreversible [12–13]. Aconitase inhibition was caused by the removal of non-hem iron ions from Fe-S clusters in the enzyme active center [12–13, 62]. The existence of such mechanism in cells *in situ* is confirmed here, by finding that each of the VGCC antagonist, partially alleviated inhibition of aconitase activity by limiting Zn^2+^ uptake (Fig 4C, Table 2). On the other hand, Zn^2+^-evoked inhibition of IDH-NADP activity was suggested as being caused by its competitive interaction with magnesium cations in the enzyme active center [63]. However, our results suggest that *in situ* inhibition of IDH-NADP by accumulated Zn^2+^ was overcome only by nifedipine. Nifedipine is the strongest L-type VGCC antagonist with pleiotropic, antioxidative properties preventing TBARS overproduction (Fig 4B and 4D–4F, Table 2)[64–65]. On the other hand, ω-conotoxins, which were devoid of such bio-activity, failed to alleviate Zn^2+^ evoked IDH-NADP inhibition (Fig 4A–4F, Table 2). (Fig 4A–4F, Table 2). This indicates that Zn^2+^-induced inhibition of IDH-NADP activity was not triggered directly by Zn^2+^ itself, but by its promotion of oxidative stress (Table 2, Fig 4D)[9, 66–67]. These suggestions are confirmed here by (i) the existence of an inverse correlation between IDH-NADP activity and alterations in TBARS accumulation, as well as by a lack of correlation with intracellular Zn^2+^ load (Fig 4D), (ii) Zn^2+^[IC_**50**_] against IDH-NADP, being 9 times higher than against aconitase (Fig 2D and 2F), (iii) the existence of an inverse correlation between aconitase activity and Zn^2+^ accumulation in SN56 cells and the absence thereof with TBARS accumulation, respectively (Fig 4B and 4C).

Thereby, the potencies of specific VGCC inhibitors in alleviating the cytotoxic effects of Zn^2+^ may reflect the potential, fractional contribution of each channel to Zn^2+^ neuropathology (Table 2) [21]. These protective effects were demonstrated by the capacity of particular VGCCs to alleviate Zn^2+^-evoked inhibition of PDHC, aconitase and IDH-NADP activities (Table 2, Fig 4A–4F). The respective significant correlation plots support the assumption that the neuroprotective effects of VGCCs antagonists were predominately linked with an alleviation of intracellular Zn^2+^ overload and/or TBARS overproduction (Table 2, Fig 4A–4F).

The presented data demonstrate that VGGCs facilitate Zn^2+^ and Ca^2+^ entry to chronically depolarized neurons. An excess of Zn^2+^ in their intracellular compartments: (i) inhibits PDHC limiting provision of acetyl-CoA for the TCA cycle in their mitochondria (ii) obstructs its metabolic flow through the TCA cycle inhibiting aconitase and IDH-NADP (iii) yields suppression of ATP production activating free radical synthesis and GSH oxidation (iv) that results in loss of neuronal viability (Table 2, Figs 3A, 3C and 4A–4F)[68]. These data indicate that Zn^2+^ overload through VGGs may be the earliest neurotoxic signal affecting multiple metabolic steps crucial for neuronal functions and survival. Rapid reversal of these effects through Zn^2+^ removal may be important for cell recuperation during recovery after excitotoxic-events.

## Supporting information

S1 Fig The concentration-dependent influence of calcium channel antagonists on intracellular levels in SN56 cells treated for 30 min by 0.15 mmol/L Zn^2+^. (A) Ca^2+^ and (B) Zn^2+^. Data are means ± SEM from 3–9 experiments. Significantly different from SN56 control (*p<0.05, **p<0.01, ***p<0.0001).
(TIF)

## Abbreviations

Aco: aconitase
AD: Alzheimer’s disease
GVIA: ω-conotoxin GVIA
GSH: reduced glutathione
GSSG: oxidized glutathione
IDH: isocitrate dehydrogenase
MVIIC: ω-conotoxin MVIIC
NF: nifedipine
TBARS: thiobarbituric acid reactive substances
TCA: tricarboxylic acid cycle
VGCC: voltage gated calcium channels
