# Supplementary figures and images for: Protective effects of voltage-gated calcium channel antagonists against zinc toxicity in SN56 neuroblastoma cholinergic cells
Source: PLoS One. 2018 Dec 20;13(12):e0209363. doi: 10.1371/journal.pone.0209363 (PMC6301650; doi:10.1371/journal.pone.0209363)

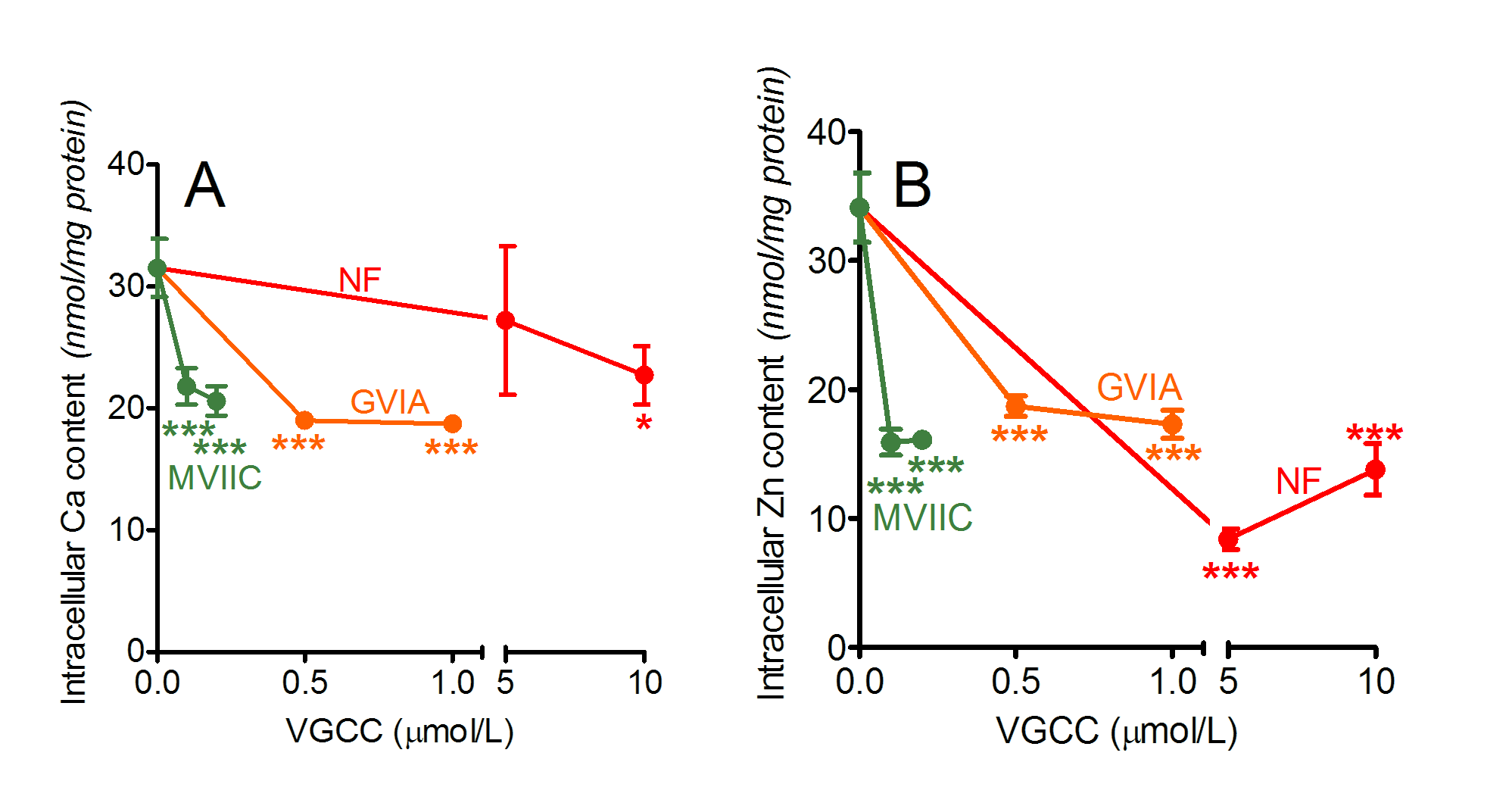

Supplement: S1 Fig — (A) Ca2+ and (B) Zn2+. Data are means ± SEM from 3–9 experiments. Significantly different from SN56 control (*p<0.05, **p<0.01, ***p<0.0001). (TIF) [file pone.0209363.s001.tif]
